# Interfacial Microstructure and Mechanical Properties of 1Cr18Ni9Ti/1Cr21Ni5Ti Stainless Steel Joints Brazed with Mn-Based Brazing Filler

**DOI:** 10.3390/ma15197021

**Published:** 2022-10-10

**Authors:** Lei Chen, Huize Chen, Weipeng Yang, Qinlian Zhang, Bo Yang, Yazhen Hu, Xiaoqing Si, Tong Lin, Jian Cao, Junlei Qi, Chun Li

**Affiliations:** 1 State Key Laboratory of Advanced Welding and Joining, Harbin Institute of Technology, Harbin 150001, China; 2 Xi’an Space Engine Company Limited, Xi’an 710021, China

**Keywords:** 1Cr18Ni9Ti, 1Cr21Ni5Ti, Mn-based brazing filler, microstructure, diffusion activation energy

## Abstract

The problem of stainless steel brazing is still the focus of scientific research. In this work, the Mn-based brazing filler was used to braze 1Cr18Ni9Ti and 1Cr21Ni5Ti stainless steel. The typical microstructure of the 1Cr18Ni9Ti/1Cr21Ni5Ti joint was analyzed in detail, and the interface structure of the joint was determined to be 1Cr18Ni9Ti/Mn(s, s)/1Cr21Ni5Ti. The brazing temperature and holding time were shown to have a great influence on the microstructure of the brazed joint. The tensile strength of brazed joints first increased and then decreased with the rising of the brazing temperature and the holding time. The maximum tensile strength was 566 MPa when the joints were brazed at 1125 °C for 15 min. The diffusion of Mn and Cr was an important factor affecting the quality of the joints. The diffusion distances of Mn and Cr at different brazing temperatures and holding times were measured, and the diffusion activation energy and diffusion coefficient were achieved by the Arrhenius equation.

## 1. Introduction

Stainless steels are widely used in aerospace, automotive, and chemical industries due to their high strength, good oxidation resistance, and corrosion resistance [1,2,3,4]. With the rapid development of technology and industry, welding and joining are applied to make complex stainless steel structures in order to meet pluralistic usage requirements [5,6,7,8]. Stainless steel joints can be widely found in core panels, heat exchangers, and metal supports which are more and more used in buildings, automobile exhaust systems, and fuel cells [9,10,11,12]. Compared to other joining methods, brazing is a joining method in which the base materials and the brazing filler (below the melting point of the base materials) are heated to the melting temperature of the brazing filler together, and then the liquid brazing filler is used to fill the brazing seam to join the base materials, so it has less impact on the properties of the base material and is very suitable for the joining of compact structures [13,14,15]. The brazing of stainless steel has been extensively studied. The commonly used filler alloys for brazing stainless steel are Ag-based [16,17], Cu-based [5,18,19], Ni-based [13,20,21], and Mn-based [22,23] brazing fillers. However, the brazed joint is still the weakest part of the whole component and sometimes the brazed structure could undergo unexpected failure. For example, there are intermetallic compounds in the brazed seam and the voids will appear in it due to unsuitable parameters [24,25,26], thus the defects in the joints can expand rapidly after the high temperature and high strain processes [27,28]. Among them, Mn-based brazing filler can meet the high-temperature service conditions of stainless steel brazed joints due to its good weldability and excellent high-temperature mechanical properties [29]. Zhen et al. [22] successfully brazed 1Cr17Ni2 stainless steel and QAl7 aluminum bronze with Cu-Mn-Ni-Ag brazing filler. The results of the experiment showed that the brazed seam was mainly composed of a solid solution phase. The interdiffusion of elements between the base material and the brazing filler leads to strong interfacial bonding, which makes the 1Cr17Ni2/QAl7 brazed joints have high mechanical properties. At the same time, studies have shown that the diffusion of elements has a great relationship with the strength of brazed joints [16,30]. Therefore, it is necessary to understand the diffusion behavior of atoms during the brazing. Diffusion activation energy (*Q*) and the diffusion coefficient (*D*) are physical quantities that describe how easily atoms can diffuse [31,32,33]. Theoretical analysis and experiments show that there is a semi-empirical correlation between the *Q* and the pre-exponential factor in the Arrhenius equation of the diffusion equation [34,35,36,37]. Hence, this makes it possible to analyze the degree of diffusion of atoms in brazed joints.

In this paper, Mn-based brazing filler (BMn70NiCr) was used to braze 1Cr21Ni5Ti and 1Cr18Ni9Ti stainless steels. The microstructure of the brazed joint was analyzed in detail, and the microstructure evolution model was established. The effects of brazing process parameters on the microstructure and mechanical property of the joints were investigated, and the fracture modes of the joints were studied. The diffusion distances of Mn and Cr were measured by line scan results, and the diffusion coefficients and diffusion activation energies were calculated by the Arrhenius equation.

## 2. Materials and Experimental Procedures

The base materials 1Cr18Ni9Ti and 1Cr21Ni5Ti stainless steels were purchased from Hebei Luo Hong Technology Co., Ltd, Hebei province, China. The tensile strengths of 1Cr18Ni9Ti and 1Cr21Ni5Ti were 600 MPa and 650 MPa, respectively. The brazing filler was a commercial Mn-based filler (BMn70NiCr), and the thickness was 100 μm. The chemical composition of each material is displayed in Table 1. The two stainless steels were both processed into small blocks of 5 mm × 5 mm × 2 mm and 10 mm × 10 mm × 2 mm using wire electrical discharge machining (WEDM) for the observation of the microstructure and the measurement of the element diffusion distance. Then the surface of the small pieces was polished to 2.5 μm. The brazing filler was cut to a size of 5 mm × 5 mm. Finally, the stainless steel blocks and the brazing filler pieces were ultrasonically cleaned in ethanol for 10 min.

The brazing process was carried out in a vacuum furnace. The brazing joints were assembled into two sandwich structures, one of them from bottom to top was 1Cr21Ni5Ti, BMn70NiCr brazing filler, and 1Cr18Ni9Ti, the other was 1Cr18Ni9Ti, BMn70NiCr brazing filler, and 1Cr21Ni5Ti. The sizes of the stainless steel at the bottom was 10 mm × 10 mm, and the top was 5 mm × 5 mm. A pressure of 6 KPa was applied on the surface of the sample to ensure reliable contact between the base materials and the brazing filler. During the brazing process, the heating rate was 20 °C/min until the set brazing temperature and then it was held for different times. The cooling rate was 15 °C/min.

The microstructures of the brazed joints were analyzed via scanning electron microscopy (SEM, Quanta 200 FEG, ThermoFisher, Hillsboro, OR, USA) equipped with energy dispersive spectrometer (EDS). First, the brazed specimen was cut along the vertical brazed seam, and then the cutting surface was polished to 1 μm. The EDS result is the average value of three points in the same area of the sample. Diffusion distances were determined by EDS line scan. The surface of the larger stainless steel was the initial diffusion boundary, and the stable point of the element atomic percentage was the end of diffusion. The results of ten positions were averaged as the diffusion distance. The reaction phases in the joints were identified by X-ray diffractometer (XRD, D8-Advance, Bruker, Karlsruhe, Germany) with Cu-Kα radiation.

The mechanical properties of the joints were characterized by the tensile strength tests using an electronic universal mechanical testing machine (Instron 5569, Instron, Boston, MA, USA). The two stainless steels were both cut into blocks of 15 mm × 15 mm × 15 mm for brazing and then the tensile test was conducted. The brazing filler was cut to a size of 15 mm × 15 mm. The pretreatment and the assembly structure of the tensile specimen are the same as the microstructure specimen. After the brazing, the brazed specimen was processed into I-shape with a size of 9 mm × 30 mm × 2 mm, the length of the parallel area was 14 mm and the width was 2 mm, the circular arc radius on both sides was 2 mm. The tests were performed at room temperature with a loading rate of 2 mm/min. The final data were the average of five specimens under the same parameter to ensure accuracy.

## 3. Results and Discussion

### 3.1. Typical Microstructure of the 1Cr21Ni5Ti/1Cr18Ni9Ti Joint Brazed Using BMn70NiCr Filler

**Figure 1:**
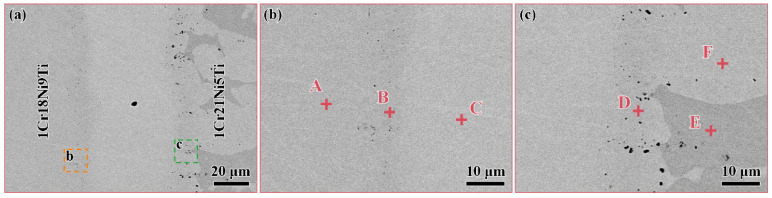
(**a**) The SEM images of 1Cr18Ni9Ti and 1Cr21Ni5Ti stainless steels brazed with BMn70NiCr filler at 1175 °C for 30 min and enlarged images of (**b**) the 1Cr18Ni9Ti side, Points A–C are the positions of EDS analysis at 1Cr18Ni9Ti, the interface and the brazed seam; (**c**) the 1Cr21Ni5Ti side, Points D–F are the positions of EDS analysis at the interface, the austenite and the ferrite in 1Cr21Ni5Ti respectively.

Since the solidus and liquidus lines of the BMn70NiCr brazing filler are 1035 °C and 1080 °C, the brazing temperature range was determined to be 1100 °C to 1200 °C. The parameters were set every 25 °C and a total of six groups. The holding time was first set to 30 min in order to ensure that the liquid brazing filler fully filled the brazing seam and the diffusion process was thoroughly carried out. Figure 1 shows the microstructure and enlarged images of the 1Cr18Ni9Ti/1Cr21Ni5Ti joint brazed with BMn70NiCr filler at 1175 °C for 30 min. It can be observed that the brazing filler achieved a good metallurgical bond with the base materials. The stainless steel on the right showed two different contrasts, which should be 1Cr21Ni5Ti duplex steel, and there was only one contrast phase in the brazed seam. The width of the brazed seam was about 60 μm, and there were no obvious defects such as cracks or voids in it.

The EDS analysis of the phases that are marked in Figure 1 A–F is displayed in Table 2. The results show that the main elements in the base materials on both sides were Fe, Cr, and Ni, and the main elements in the brazed seam were Mn, Fe, Cr, and Ni. Among them, the content of Cr in the phases A and D was less than that in the phase C, indicating that the phases A and D should be austenite, and the phase C was ferrite. The phase C in the brazed seam contained much Mn, indicating that it was a Mn-based solid solution. The content of Fe at the interface between the base materials and the brazed seam on both sides was higher than that in brazed seam, so phases B and D should be a Fe-based solid solution. Comparing the chemical composition of the brazed seam and the initial brazing filler, it can be seen that the Fe in the brazed seam was completely derived from the dissolution of the base materials, and a small amount of Cr and Ni dissolved into the brazed seam as well. At the same time, the Mn in the brazing filler also diffused into the base materials on both sides.

**Figure 2:**
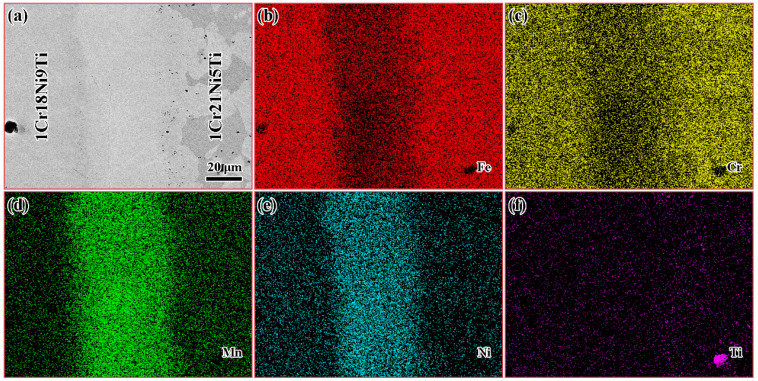
(**a**) The SEM image and the corresponding element map distribution of 1Cr18Ni9Ti/BMn70NiCr/1Cr21Ni5Ti joint at 1175 °C/30 min: (**b**) distribution of Fe; (**c**) distribution of Cr; (**d**) distribution of Mn; (**e**) distribution of Ni; (**f**) distribution of Ti.

The EDS mapping was performed on the brazed joint to clarify the element distribution, and the results are shown in Figure 2. Fe and Cr were concentrated in the base materials on both sides, and a small amount in the brazed seam. There were a lot of Mn and Ni in the brazed seam, but less distribution in the base materials.

**Figure 3:**
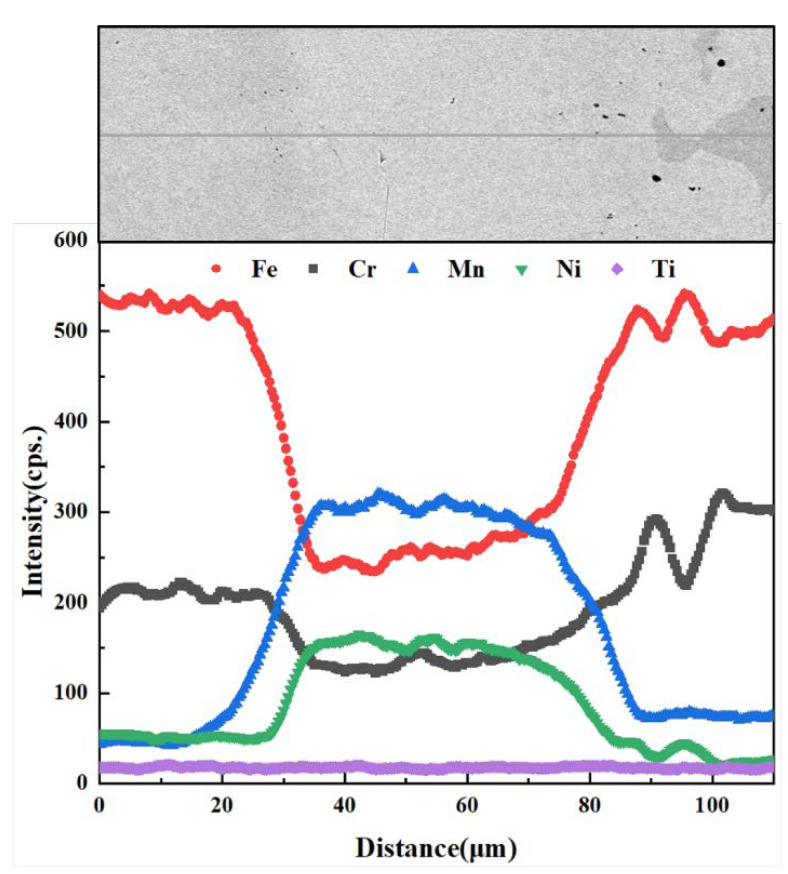
The SEM image and the corresponding element EDS line scans of 1Cr18Ni9Ti/BMn70NiCr/1Cr21Ni5Ti joint at 1175 °C/30 min.

The EDS line scan analysis of the brazed joint was further carried out, which is shown in Figure 3. It was found that the elements in the base materials and the brazing filler diffused to each other. Mn and Ni diffused into the base materials, forming the diffusion reaction layers on both sides. The diffusion distance of Mn (~30 μm) in 1Cr18Ni9Ti was greater than that of Cr (~20 μm) in 1Cr18Ni9Ti, while the diffusion distance of the two elements (~30 μm) in 1Cr21Ni5Ti was roughly equal.

**Figure 4:**
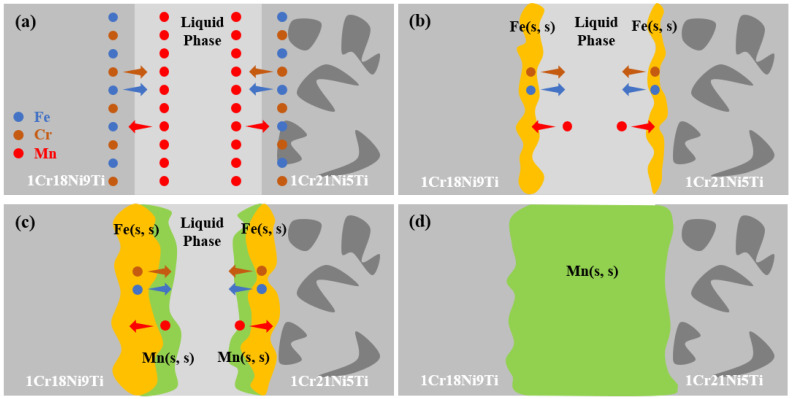
The interface formation model: (**a**) the brazing filler melted to form the liquid phase; (**b**) the elements in the stainless steel dissolved into the brazing filler; (**c**) the holding stage; (**d**) the final morphology of the as-brazed joint.

The interface formation model shown in Figure 4 can explain the possible bonding behavior during the brazing process. First, the brazing filler melted to form a liquid phase. The base materials began to dissolve into it and changed the chemical composition of the molten filler, as shown in Figure 4a. Then, the elements between the brazing filler and the base materials diffused to each other, and the Fe-Mn-Cr solid solution was formed at the solid–liquid interface [23,38,39]. Later, with the decrease of the temperature, the liquid phase in the brazed seam decreased, and the Mn-based solid solution was gradually formed along the solid–liquid interface [40,41]. According to the Fe-Mn phase, the diagram [42] shows that Fe and Mn had a large solid solution interval, as the content of Fe increased, the melting temperature of the molten filler also increased. Because of Fe continuously dissolving from the base materials into brazing filler, the brazed seam gradually solidified during the holding stage [29], as Figure 4c shows. Finally, the Fe, Mn, Cr, and Ni in the brazing filler and the base material further diffused and formed a continuous Mn-based solid solution in the brazed seam. Hence, the microstructure of the brazed joint was 1Cr21Ni5Ti/Mn(s, s)/1Cr18Ni9Ti.

### 3.2. Effect of Brazing Temperature on the Microstructure and Mechanical Properties of 1Cr21Ni5Ti/BMn70NiCr/1Cr18Ni9Ti Joints

**Figure 5:**
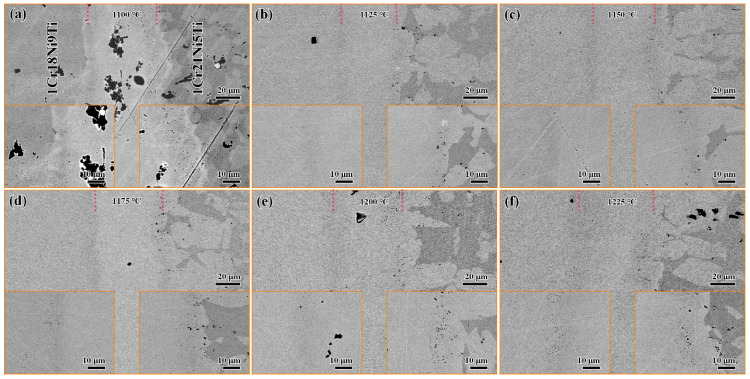
The SEM images of 1Cr18Ni9Ti and 1Cr21Ni5Ti stainless steels brazed with BMn70NiCr filler at different temperatures for 30 min: (**a**) 1100 °C; (**b**) 1125 °C; (**c**) 1150 °C; (**d**) 1175 °C; (**e**) 1200 °C; (**f**) 1225 °C.

The 1Cr18Ni9Ti/BMn70NiCr/1Cr21Ni5Ti joints were brazed at different brazing temperatures (1100 °C, 1125 °C, 1150 °C, 1175 °C, and 1120 °C) for 30 min to investigate the effect of the brazing temperature on the microstructure and mechanical properties. As shown in Figure 5, when the brazing temperature was low (1100 °C), there were a few voids in the brazing seam. The reason is that the solidus and liquidus lines of the brazing filler were 1035 °C and 1080 °C, at this temperature, the brazing filler melts poorly, and the gas in the brazed seam cannot escape in time. Similar situations have been reported in Reference [43]. When the brazing temperature reached 1125 °C, the voids in the brazed seam disappeared. Then, with the increase of the brazing temperature, the diffusion rate of the elements at the interface increased, and the width of the brazed seam increased continuously.

**Figure 6:**
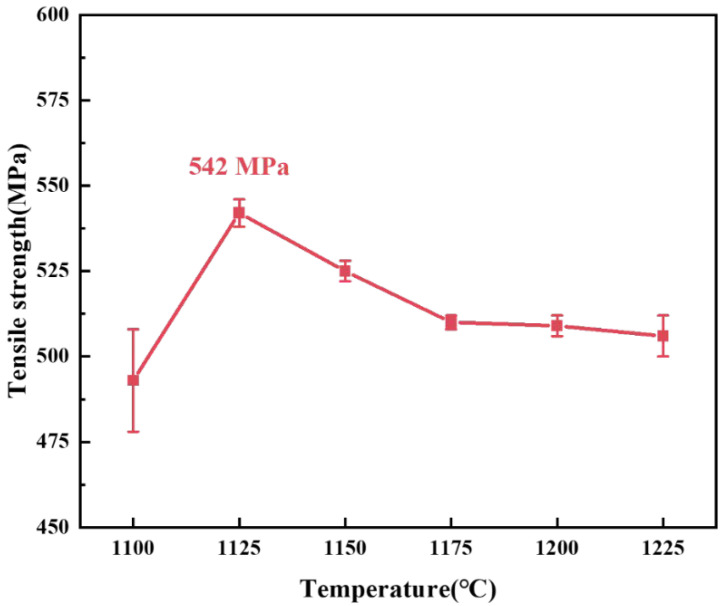
The tensile strength of 1Cr18Ni9Ti/BMn70NiCr/1Cr21Ni5Ti joints at different brazing temperatures.

The tensile test results of the brazed joints in different temperatures are shown in Figure 6. With the increase of the brazing temperature, the tensile strength of the brazed joints first increased and then decreased, and the maximum tensile strength was 542 MPa when the joints were brazed at 1125 °C for 30 min. When the brazing temperature was low, there were voids in the brazed seam, resulting in the low strength of the brazed seam. As the brazing temperature increased, the fluidity of the brazing filler increased. The diffusion of the elements in the base materials and the brazing filler accelerated, the voids in the brazing seam disappeared, and the strength of the brazed seam was improved. Then the fracture was transferred from the brazed seam to the 1Cr18Ni9Ti stainless steel side, and the tensile strength of the joints increased. However, when the brazing temperature id too high, the grains of the stainless steel grow and its strength decreases [44,45,46], so the tensile strength of the joints decreases.

### 3.3. Effect of Holding Time on the Microstructure and Mechanical Properties of 1Cr21Ni5Ti/BMn70NiCr/1Cr18Ni9Ti Joints

**Figure 7:**
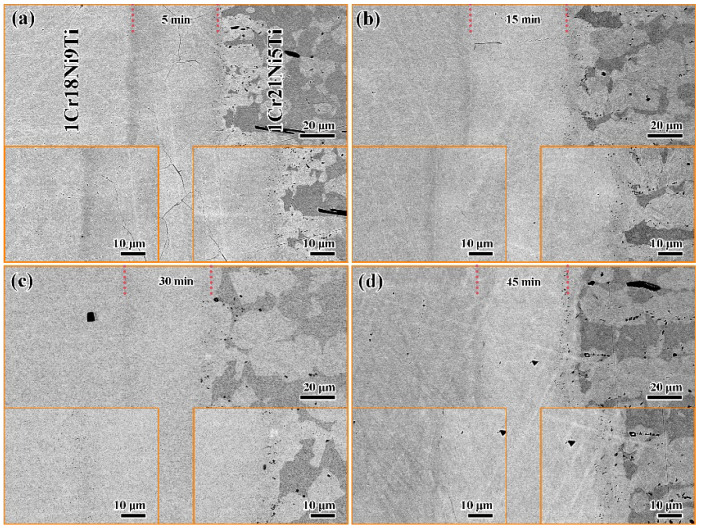
The SEM images of 1Cr18Ni9Ti and 1Cr21Ni5Ti stainless steels brazed with BMn70NiCr filler at 1125 °C for different holding times: (**a**) 5 min; (**b**) 15 min; (**c**) 30 min; (**d**) 45 min.

Since the joints had the highest tensile strength when brazed at 1125 °C, the effect of holding time on the microstructure and mechanical properties of 1Cr18Ni9Ti/BMn70NiCr/1Cr21Ni5Ti joints was further studied by setting different holding times (5 min, 15 min, 30 min, and 45 min) at 1125 °C, which is shown in Figure 7. When the holding time was short (5 min), there were a few cracks in the brazed seam. This is because the elements in the base materials and brazing filler diffuse unevenly, as the temperature decreases, the low melting point filler solidifies and shrinks, which cannot fill the brazed seam well, resulting in cracks. When the holding time reached 15 min, the cracks in the brazed seam disappeared. The width and structure of the brazed seam did not change significantly, indicating that the diffusion of elements between the base materials and the brazing filler reached equilibrium after holding for 15 min.

**Figure 8:**
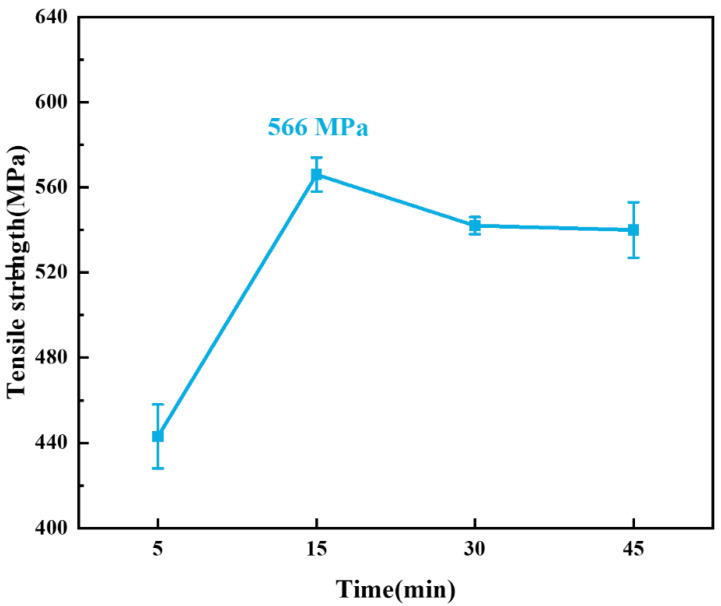
The tensile strength of 1Cr18Ni9Ti/BMn70NiCr/1Cr21Ni5Ti joints at different holding times.

The tensile test results of brazed joints with different holding times are shown in Figure 8. As the holding time extended, the tensile strength of brazed joints increased first and then decreased, and the maximum tensile strength was 566 MPa when the joints were brazed at 1125 °C for 15 min. When the holding time was short (5 min), the diffusion degree of Mn, Cr, and Fe in the base material and the brazing filler was low, so that the interface bonding between the base material and the brazing filler was weak. At the same time, solidification cracks appeared in the brazed seam due to inhomogeneous diffusion, so that the strength of the brazed seam was poor. Both of them led to the lower tensile strength of the brazed joints. With the extension of the holding time (15 min), the diffusion of Mn, Cr, and Fe was increased and more uniform. Then the interface bonding was better and the cracks in the brazed seam disappeared, so that the strength of the brazed seam was improved and the tensile strength of the joints increased to 566 MPa. However, long holding time will also make the grains of the stainless steels grow, reducing the tensile strength of the joints.

### 3.4. Fracture Analysis

**Figure 9:**
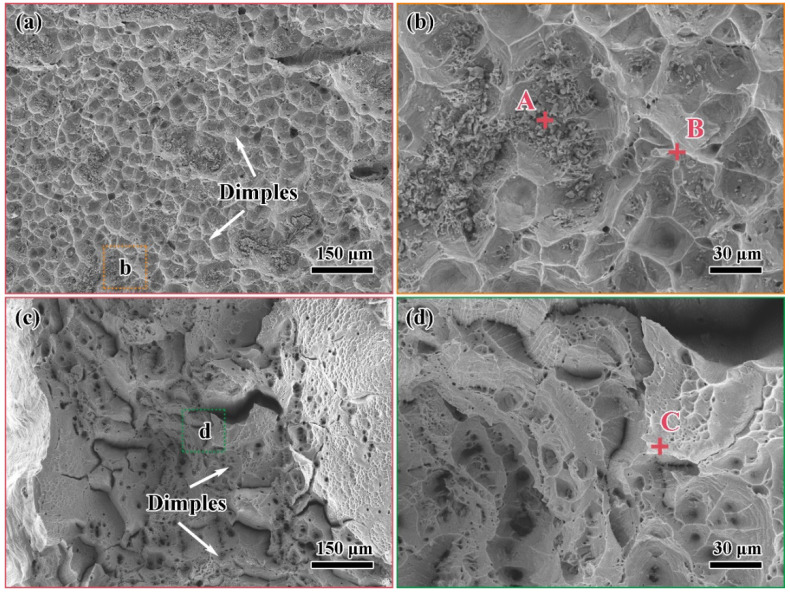
The fracture SEM images of 1Cr18Ni9Ti/BMn70NiCr/1Cr21Ni5Ti joints at different parameters: (**a**) 1100 °C/30 min; (**b**) enlarged image of (**a**), Points A and B are the positions of EDS analysis at the pits and the bulges respectively; (**c**) 1125 °C/30 min; (**d**) enlarged image of (**c**), Point C is the position of EDS analysis at the fracture.

**Figure 10:**
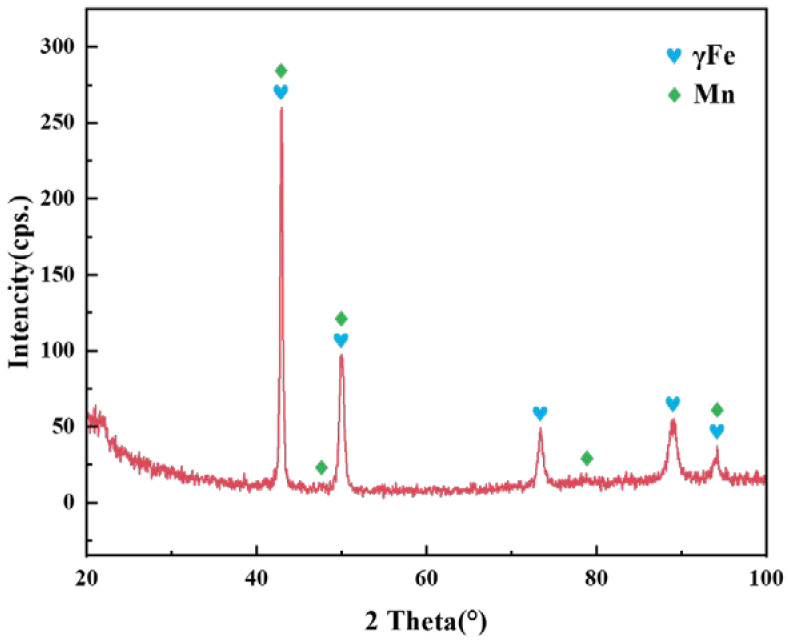
The XRD pattern of the fracture at 1100 °C/30 min.

Figure 9 shows the tensile fracture of the brazed joint under the parameters of 1100 °C/30 min and 1125 °C/30 min, respectively. They represent two typical fracture modes in tensile experiments. The EDS results of the marked points are displayed in Table 3. Figure 9a,b shows the result when the joints fractured from the brazed seam at 1100 °C/30 min. In addition to that, the same fracture behavior occurred at 1125 °C/5 min. Although the fracture surface was relatively flat macroscopically, the microstructure was uneven with numerous dimples. The EDS results of points A and B show that the fracture contained much Mn. Combined with the XRD pattern of Figure 10, the phase of the fracture was a Mn-based solid solution. This result verifies the microstructure of the brazed joint in Section 3.2 and Section 3.3. When the brazing temperature was low or the holding time was short, the elements’ diffusion between the base materials and the brazing filler was less, voids or solidification cracks appeared in the brazed seam, and the fracture was located in it. Despite the Mn-based solid solution undergoing plastic deformation under the action of tensile stress, its strength and toughness were not as good as the base materials. Only 1Cr18Ni9Ti had slight necking. The break elongation of the brazed joints was less than 20%, which was lower than that of 1Cr18Ni9Ti itself. Figure 9c,d shows the result when the joints fractured from the 1Cr18Ni9Ti at 1125 °C/30 min. This fracture mode occurred when the brazing temperature was higher than 1125 °C and the holding time was longer than 15 min. The brazed joints were necked significantly and the fracture had obvious plastic deformation. There were a large number of dimples on the fracture surface. The EDS results at point C show that the fracture contained more Fe and Cr. Due to a large amount of Fe and Cr in the base materials dissolving into the brazing filler, the strength of the brazed seam was greater than that of 1Cr18Ni9Ti, and the fracture occurred on the side of 1Cr18Ni9Ti. The break elongation of the brazed joints exceeded 50%, which was equivalent to that of 1Cr18Ni9Ti.

### 3.5. Diffusion Behavior of Mn and Cr in 1Cr21Ni5Ti/BMn70NiCr/1Cr18Ni9Ti Joints

Mn and Cr were the main elements in the brazing filler and stainless steel base materials, respectively. Hence, it was necessary to investigate their diffusion in the brazed joint. By measuring the diffusion distances of Mn and Cr at different brazing temperatures and holding times, the corresponding diffusion coefficients and diffusion activation energies could be calculated to better control the brazing process parameters. However, measuring the diffusion distances of different parameters is rather cumbersome. The calculation of the diffusion coefficient and diffusion activation energy can help us understand the diffusion mechanism of Mn or Cr and to better control the brazing process parameters. It can also guide us to estimate the parameters faster in the actual engineering requirements.

According to the empirical formula, the relationship between the elements’ diffusion distance (*δ*) and time (*t*) can be written as [31,32,47]:

*δ*^n^ = *Dt* (1)

In Formula (1), *δ* is the average diffusion distance of Mn or Cr (m), n is the time exponent, *D* is the diffusion coefficient (m^2^/s), and *t* is the holding time of this experiment (s).

The Arrhenius equation describes the relationship among diffusion coefficient (*D*), diffusion activation energy (*Q*), and temperature (*T*) [35,41]:

*D* = *D*_0_e^−*Q*/*RT*^ (2)

In Formula (2), *D*_0_ is the pre-exponential factor (m^2^/s) and it is only affected by the element itself, *Q* is the diffusion activation energy (J/mol) and it can be regarded as a constant in the range of the brazing temperature, *R* is the gas constant (8.314 J/(mol·K)), *T* is the brazing temperature (K).

Substitute Formula (2) into (1), we obtain:

*δ*^n^ = *D*_0_*t*e^−*Q*/*RT*^ (3)

Take the logarithm of Formula (3), we get:

nln*δ* = −*Q*/*RT* + ln*D*_0_*t* (4)

According to Formula (4), if the brazing temperature or the holding time is fixed, then the diffusion activation energy (*Q*) and pre-exponential factor (*D*_0_) of Mn or Cr in 1Cr18Ni9Ti and 1Cr21Ni5Ti stainless steels can be obtained.

**Figure 11:**
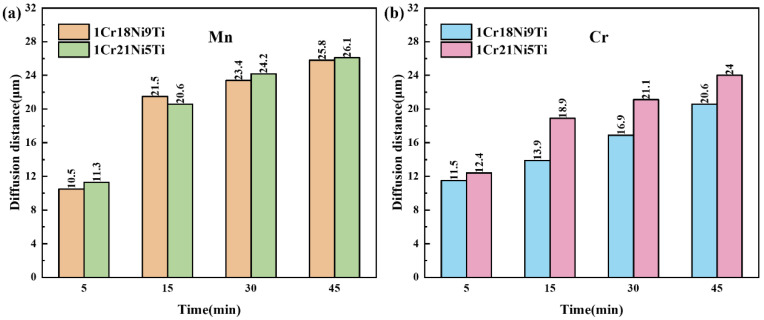
Diffusion distances of (**a**) Mn and (**b**) Cr in 1Cr18Ni9Ti and 1Cr21Ni5Ti stainless steel at different holding times.

Figure 11 shows the evolution of the diffusion distance of Mn and Cr in 1Cr18Ni9Ti and 1Cr21Ni5Ti stainless steels after being brazed at 1125 °C for different holding times. With the increase of the holding time, the diffusion distances of Mn and Cr in the base materials increase, and the diffusion distance of Cr in 1Cr21Ni5Ti is always greater than that in 1Cr18Ni9Ti.

**Figure 12:**
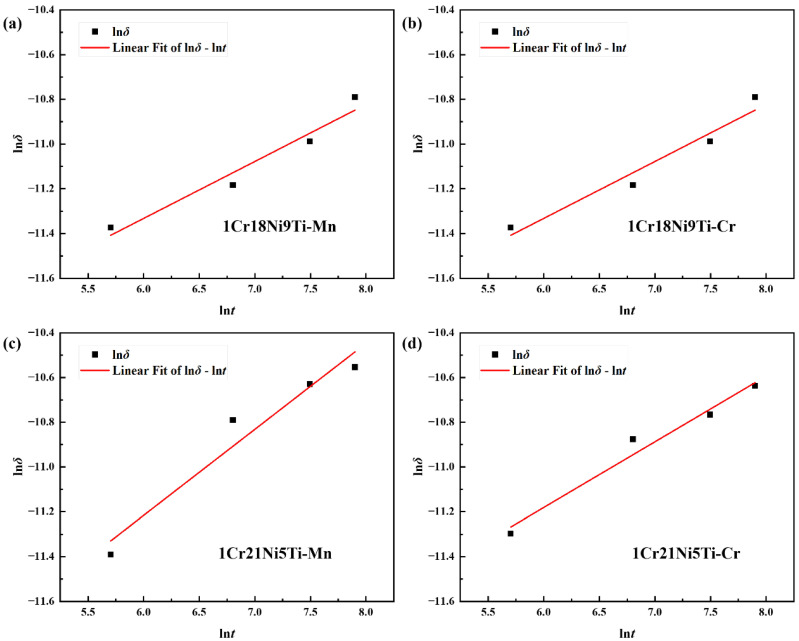
The fitted ln*δ*-ln*t* curves of diffusion distances of (**a**) Mn in 1Cr18Ni9Ti; (**b**) Cr in 1Cr18Ni9Ti; (**c**) Mn in 1Cr21Ni5Ti; (**d**) Cr in 1Cr21Ni5Ti.

Keeping the brazing temperature as a constant, it can be found that ln*δ* and ln*t* have a linear relationship, and the inverse of the time exponent (1/n) is the slope of the line. Take ln*t* as the abscissa and ln*δ* as the ordinate for linear fitting, the result is shown in Figure 12. Then, we can obtain the time exponent of the diffusion distance of Mn in stainless steels is about 2.5, and the time exponent of Cr is about 4.

**Figure 13:**
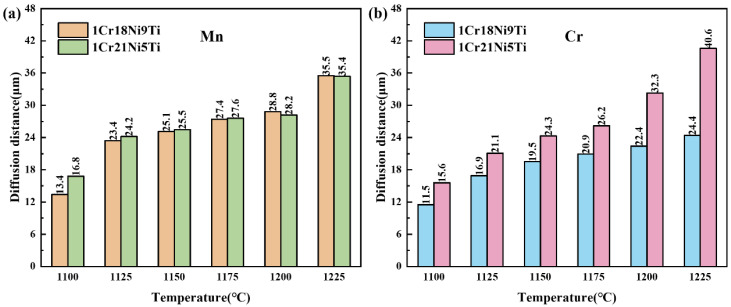
Diffusion distances of (**a**) Mn and (**b**) Cr in 1Cr18Ni9Ti and 1Cr21Ni5Ti stainless steel at different brazing temperatures.

Figure 13 shows the evolution of the diffusion distances of Mn and Cr in 1Cr18Ni9Ti and 1Cr21Ni5Ti stainless steel after holding for 30 min at different temperatures. With the increase of temperature, the diffusion distances of Mn and Cr in the base materials increase, and the increase of the diffusion distance of Cr element in 1Cr21Ni5Ti is greater than that in 1Cr18Ni9Ti.

**Figure 14:**
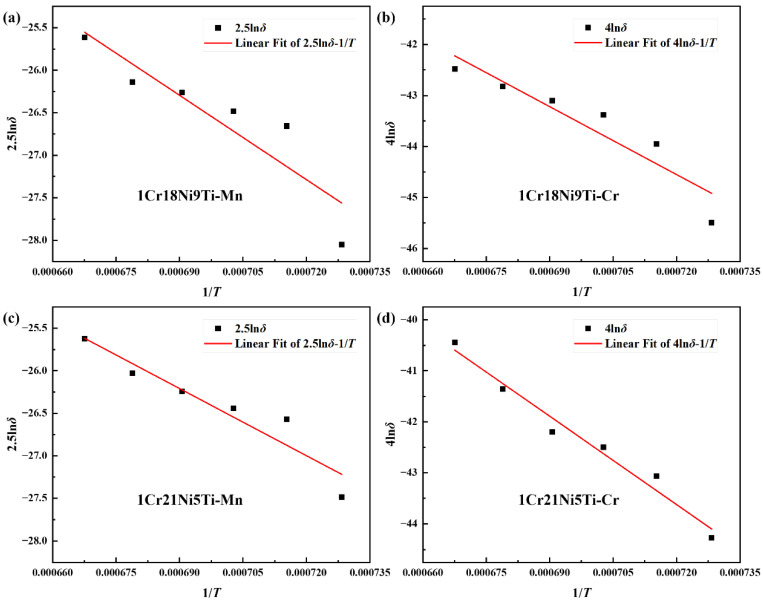
The fitted nln*δ*-1/*T* curves of diffusion distances of (**a**) Mn in 1Cr18Ni9Ti; (**b**) Cr in 1Cr18Ni9Ti; (**c**) Mn in 1Cr21Ni5Ti; (**d**) Cr in 1Cr21Ni5Ti.

Keeping the holding time as a constant, it can be found that nln*δ* and 1/T have a linear relationship, the slope of the line is −*Q*/R and the intercept is ln*D*_0_*t*. Take 1/T as the abscissa and nln*δ* as the ordinate for linear fitting, the result is shown in Figure 14. Through calculating, the pre-exponential factor and diffusion activation energy of Mn in 1Cr18Ni9Ti are *D*_0_(Mn) = 1.38 × 10^−4^ m^2^/s and *Q*(Mn) = 1.10 × 10^5^ J/mol, and in 1Cr21Ni5Ti are *D*_0_(Mn) = 2.23 × 10^−5^ m^2^/s and *Q*(Mn) = 8.76 × 10^4^ J/mol. The pre-exponential factor and diffusion activation energy of Cr on 1Cr18Ni9Ti side are *D*_0_(Cr) = 2.37 × 10^−5^ m^2^/s and *Q*(Cr) = 9.22 × 10^4^ J/mol, and on 1Cr21Ni5Ti side are *D*_0_(Cr) = 3.30 × 10^−4^ m^2^/s and *Q*(Cr) = 1.20 × 10^5^ J/mol.

When comparing the diffusion activation energies of Mn and Cr in fcc-Fe with Reference [37], the diffusion activation energies measured in this work were smaller but the same order of magnitude, which may be the formation of the liquid phase during the brazing process made the diffusion of Mn and Cr easier. However, the pre-exponential factor (*D*_0_) of Mn was higher than that of Reference [48], and Cr was lower. Comparing the ratio of elements in the base materials to the brazing filler, it can be found that the difference in atomic concentration was the main driving force for the diffusion of Mn or Cr.

According to Formula (2), the diffusion coefficients of Mn and Cr on 1Cr18Ni9Ti side at 1125 °C are *D*(Mn)= 1.07 × 10^−8^ m^2^/s and *D*(Cr)= 8.51 × 10^−9^ m^2^/s, and on 1Cr21Ni5Ti side are *D*(Mn)= 1.19 × 10^−8^ m^2^/s and *D*(Cr)= 1.08 × 10^−8^ m^2^/s.

## 4. Conclusions

In this experiment, 1Cr18Ni9Ti and 1Cr21Ni5Ti were brazed with Mn-based brazing filler. The microstructure and mechanical properties of the 1Cr18Ni9Ti/BMn70NiCr/1Cr21Ni5Ti joints were studied in detail, and the diffusion behavior of two main elements Mn and Cr in the stainless steels was investigated. The conclusions are summarized as follows:

(1) The 1Cr18Ni9Ti/1Cr21Ni5Ti joints were successfully brazed using BMn70NiCr filler at 1175 °C/30 min without voids and cracks. The typical microstructure in the joint was 1Cr18Ni9Ti/Mn(s, s)/1Cr21Ni5Ti.
(2) The brazing temperature, holding time, and brazing seam width affected the microstructure of the 1Cr18Ni9Ti/BMn70NiCr/1Cr21Ni5Ti joint. When the brazing temperature is low and the holding time was short, there were voids and cracks in the brazed seam due to insufficient elements’ diffusion.
(3) The tensile strengths of the brazed joint first increased and then decreased with the rising of the brazing temperature and holding time. The maximum tensile strength of the joint was 566 MPa under the parameter of 1125 °C/15 min. When the temperature was too high or the holding time was too long, the tensile strength of the joint decreased due to the grains growth in 1Cr18Ni9Ti.
(4) With the increase of the brazing temperature and the holding time, the diffusion rate and quantity of Mn or Cr increased, which led to a larger diffusion distance in the stainless steels. The formation of the liquid phase during the brazing process made the diffusion of Mn and Cr easier.
(5) The difference in atomic concentration was the main driving force for the diffusion of Mn or Cr. The diffusion activation energy of the Mn element in 1Cr18Ni9Ti was 1.10 × 10^5^ J/mol, and in 1Cr21Ni5Ti was 8.76 × 10^4^ J/mol. The diffusion activation energy of Cr element in 1Cr18Ni9Ti was 9.22 × 10^4^ J/mol, and in 1Cr21Ni5Ti was 1.20 × 10^5^ J/mol.

This work shows the effects of the brazing parameters on the microstructure and mechanical properties of the stainless steel brazing joint and could offer some guidance for future stainless steel brazing work.

## Figures and Tables

**Table 1:** Chemical composition of the stainless steel and brazing filler (wt.%).

Element	C	Si	Mn	Cr	Ni	Ti	Fe
1Cr21Ni5Ti	0.09~0.14	≤0.8	≤0.8	20~22	4.8~5.8	≤0.8	Bal.
1Cr18Ni9Ti	≤0.12	≤1.0	≤2.0	17~19	8~11	≤0.8	Bal.
BMn70NiCr	-	-	Bal.	4.5~5.5	24.0~26.0	-	-

**Table 2:** The EDS results for spots marked in Figure 1 A–F (at.%).

Element/Points	Fe	Cr	Ni	Mn	Ti
**A**	70.9	19.1	10.0	-	-
**B**	55.1	20.8	9.0	14.8	1.3
**C**	9.4	3.9	10.1	63.5	-
**D**	50.1	18.7	10.3	19.7	1.2
**E**	66.4	26.2	3.4	4.0	-
**F**	69.2	19.5	6.6	4.7	-

**Table 3:** The EDS results for spots marked in Figure 9 A–C (at.%).

Element/Points	Fe	Cr	Ni	Mn	Ti
**A**	17.7	8.0	18.8	55.0	0.5
**B**	9.4	3.9	10.1	63.5	13.1
**C**	67.2	22.7	7.3	2.3	0.5

## Data Availability

The data are not publicly available due to that it also forms part of an ongoing study.
